# Offspring defense by an urban raptor responds to human subsidies and ritual animal-feeding practices

**DOI:** 10.1371/journal.pone.0204549

**Published:** 2018-10-29

**Authors:** Nishant Kumar, Qamar Qureshi, Yadvendradev V. Jhala, Andrew G. Gosler, Fabrizio Sergio

**Affiliations:** 1 Edward Grey Institute of Field Ornithology, Department of Zoology, University of Oxford, Oxford, United Kingdom; 2 Wildlife Institute of India, Chandrabani, Dehradun, Uttarakhand, India; 3 Institute of Human Sciences, School of Anthropology and Museum Ethnography, Oxford, United Kingdom; 4 Department of Conservation Biology, Estacion Biologica de Doñana—CSIC, C/ Americo Vespucio 26, Sevilla, Spain; University of Lleida, SPAIN

## Abstract

There is a growing interest in the behavioural and life history mechanisms that allow animal species to cope with rapidly expanding urban habitats, which impose frequent proximity to humans. A particular case of behavioral bottleneck (i.e. conflicting interests) faced by animals in urban environments is how they will modulate the defence of their offspring against the potential danger represented by humans, an aspect that has received scarce research attention. We examined the nest defense against humans by a dense breeding population of a raptor, the Black Kite *Milvus migrans*, within the megacity of Delhi (India). Here, kites live on a diet dominated by human waste and meat offered through religiously motivated bird feeding practices. Nest defense levels increased with the number of offspring, and with the progression of the breeding season. Defense also intensified close to ritual-feeding areas and with increasing human waste in the streets, suggesting synergistic effects of food availability, parental investment, personality-boldness and habituation to humans, with consequent attenuation of fear. Thus, the behavioural response to a perceived threat reflected the spatial mosaic of activity of humans in the city streets, their cultural practices of ritual-feeding, and their waste-management. For synurbic species, at the higher-end spectrum of adaptation to an urban life, human cultural practices and attitudes may well be the most defining dimensions of their urban niche. Our results suggest that, after initial urban colonization, animals may continue to adapt to the typically complex, heterogeneous environments of cities through fine-grained behavioural adjustments to human practices and activities.

## Introduction

Rapid, worldwide urbanization is raising interest in urban ecology and in the ways animals adapt to novel and burgeoning urban environments [1]. In particular, behavioural mechanisms that mediate such adaptation remain an under-researched topic [2, 3], with current knowledge mostly limited to a handful of species that have only recently colonized or are in the process of capitalizing on urban environments e.g. [2, 4, 5]. Furthermore, these species have typically been studied in biogeographic regions with a long history of wildlife persecution by humans, whose proximity is frequently seen as a potential obstacle for urban colonization e.g. [6, 7]. For example, many studies have focused on flight initiation distances (FID) to explore how behavioural characteristics or personality features may allow certain individuals to better cope with proximity to humans in highly anthropogenic environments [8–12].

A different scenario may be represented by those ‘synurbic’ species [13] that have lived within human settlements for centuries. These species often show limited fear of humans and sometimes even directly exploit them, as in the case of many populations dependent on carrion or garbage in traditional human societies which, often because of the ecosystem services they provide, do not persecute them e.g. [14]. Information on the behavioural responses to humans by these urban specialists would complete our current picture of adaptation to a rapidly urbanising world and offer insights into the range of behavioural strategies potentially employed by urban wildlife to cope with a constant high proximity to humans [15, 16].

One particularly interesting case of behavioural bottleneck (i.e. conflicting interests) faced by animals in urban environments is how to modulate their defence of young in a fixed nest or den against the potential danger presented by humans. Such modulation is especially relevant for large vertebrates armed with potentially dangerous weaponry and thus theoretically capable to drive humans away. This scenario is more complex than the one examined in studies of flight initiation distance, because the fitness investment at stake (the offspring) is not mobile and cannot be fully controlled by the animal (e.g. by fleeing). Thus, it may be particularly informative of the extent to which urban animals perceive humans as a threat, how much risk they are willing to take to defend their parental investment, and how this may vary along a range of urban configurations and human attitudes towards wildlife. To our knowledge, few studies have examined such aspects and most of them have focused essentially on the comparison of behavioural traits between urban and rural individuals (review in [2, 7]). While this comparison renders important information on trait expression associated with the colonization of urban environments, it assumes that all individuals that colonized a city adopted the same behavioural strategies [16]. However, urban environments are often highly heterogeneous mosaics with marked variation in physical structure or human density [17, 18], to which animals are likely to respond, potentially selecting for a more complex array of behavioural strategies in urban animals than has thus far been recognized.

To explore these aspects, here we examine patterns of nest defense against humans by a synurbic raptor, the Black Kite *Milvus migrans*, which exploits humans for food in a megacity (Delhi, India) that incorporates a wide range of urban conditions, human densities and ritualized animal-feeding practices. The Black Kite (hereafter kite) is a medium-sized opportunistic raptor, widely distributed throughout Eurasia, Africa and Australia, and considered the most successful raptor in the world. In India, the native, resident subspecies *M*. *m*. *govinda* is synurbic (sensu [13]], occurring almost exclusively in close association with humans in towns and cities [19]. In Delhi, kites breed on both trees and artificial structures (pylons, towers), sometimes forming loose colonies and locally reaching extremely high densities, thanks to the exploitation of human food ‘subsidies’ facilitated by inefficient refuse disposal and by religious kite-feeding practices [20, 21]; (see Study area below). In particular, the relationship between kites and humans in Delhi is dual: on one hand, kites depend on humans for food and thus over-select breeding sectors in the city close to ritual-feeding sites, and with a high density of humans and of their garbage in the streets [20]. On the other hand, people sometimes rob or destroy kite nests to collect nestlings for the illegal bird trade, or to remove dangerous nest materials from electricity pylons, telephone towers or light poles during maintenance operations [22]. Thus, humans approaching a nest can be perceived by kites as a potential threat to their offspring, soliciting a defense response.

We feel that this represents a particularly interesting case study because: (1) Delhi kites directly exploit humans for food, by accessing their waste or by grabbing meat offered to them by people through religious, ritual feeding practices. Thus, they frequently come into close contact with humans, which may affect their perception and fear of humans. (2) These offerings and garbage disposal practices vary dramatically through the city (see Study area below), implying that different kites may experience and perceive people in different ways through the urban mosaic. (3) As medium-sized raptors armed with sharp talons and high aerial agility, kites are potentially well capable to inflict injury on people and drive them away from their nest-area. However, (4) much of the mortality experienced by kites is still of anthropogenic origin [22], implying a delicate trade-off between the need to come close to humans for feeding but avoid them or repel them in the appropriate context to ensure their own or their offspring safety. Given all the above, when faced with people approaching their nest, kites will need to take a quick defense-tactic decision, which may reflect these conflicting pressures. Furthermore, the balance of these pressures may change through the complex mosaic offered by this megacity of 16 million people. In particular, because ready access to dense Muslim colonies rich in ritual-subsidies is considered a key resource in this population (see [20, 21] and Study area below), we hypothesized that it could alter the profitability for kites of hygiene levels, green cover or built-up cover, ultimately affecting the defense-value of the offspring.

## Materials and methods

This research is part of a larger and long-term study on the demography of Black Kites in Delhi and has received bioethical approval by the Training, Research, and Academic Council (TRAC) of the Wildlife Institute of India, Dehradun. The project took all precautions to ensure researcher and animal safety. The study did not involve human participants other than the research team.

### Study area

Delhi is a megacity of more than 16 million inhabitants, currently covering an area of 1500 km^2^ and in constant, rapid expansion [23]. It is polycentric and heterogeneous, with a multitude of juxtaposed urban configurations, which make it difficult to establish a linear urban-rural gradient. Two aspects of Delhi are important in determining the food availability and habitat quality for kites [20]. First, large portions of the city are characterized by poor solid waste management, which affords food to kites in the form of carrion or refuse, and its associated prey-fauna (e.g. rodents, pigeons etc.). Secondly, many people (primarily in Muslim settlements) engage in the centuries-old religious practice of feeding meat scraps to kites (hereafter termed “ritualized-feeding”) typically offered by throwing meat into the air for the birds to catch. These offerings are made for a variety of reasons, such as asking for blessings and relief from sins and worries [24, 25]. Thus, waste management issues common to all communities, and cultural rituals which are more specific to some, generate spatial heterogeneity in the potential food availability for kites [20].

### Field procedures

We systematically surveyed kite nests during 2013–2016 at 24 plots of 1 km^2^, which were randomly stratified within Delhi (1500 km^2^) so as to cover all its possible urban settings, from semi-natural to extremely built-up sites (details in [21]). This resulted in a sample of 101 nests, each from a different territory, used at least once for breeding between 2013 and 2016. Nests were checked every 7–10 days until the chicks were at least 45 days old, in order to estimate the number of young raised to fledging (chicks fledge when about 48 days old) (see [21] for further details of nest checks and surveys). During each nest check, we assessed the intensity of offspring defense by the parents against our own human intrusion as follows. During each visit, a team consisting of a tree-climber (always the same for each nest) and one or two accompanying observers positioned themselves at a point from where the kite nest was in clear sight. The point was chosen so as to be clearly visible to the parent kite perched in the nest area. The team then walked slowly towards the nest. Once under the nest, we observed the behaviour of the adults for 20 minutes while the tree-climber reached the nest and checked its content. We classified the intensity of defense according to the following ordinal score: (score 0) the kite remains perched at a distance (> 20 m) or flies far away, either silently or after alarm-calling a few times; (score 1) it flies directly above the field-team in an excited manner while calling repeatedly, or perches close-by (within 20 m) and alarms continuously, or perches within a few metres of the climber (within the same nesting tree); and (score 2) it repeatedly dive-bombs at the climber and ground-team, it may even stoop among tree-branches or electricity wires, or perch a few metres from a team-member and then stoop again, sometimes hitting or scratching with open talons, while continuously alarm-calling. Thus, progressively higher scores were associated with higher costs and risks for the defending kite, including (a) increases in energy costly activities, such as alarm-calling or flapping flight, and (b) increases in potential risks, such as injuries while manoeuvring through the canopy or overhead electric wires. Throughout, the defense score refers to the maximum intensity of defense shown by either of the partners of each pair. This was justified by the fact that: (1) kites are monomorphic, making it impossible to distinguish males from females; and (2) no difference in defense levels was noticed between the two partners of a pair (if one attacked, the other also attacked, while if one remained quietly perched, the other did the same). All defense ratings were carried out between 08:00 and 18:00 hrs (local time) avoiding unusual weather conditions (e.g. rain, or excessive heat).

### Predictors of offspring defense

To investigate how kite defense varied across the Delhi mosaic of urban structure, human densities and practices, we measured a series of environmental, urban and human variables previously found to be important components of habitat quality and food availability in this population [20]. These variables are detailed in Table 1 and were devised so as to characterise: (1) the timing, context and characteristics of the defense trial (e.g. number of people in the visiting team, number of previous visits to a target nest); (2) the breeding stage, social setting (intraspecific spacing) and content of the nest during the trial (e.g. number of offspring to be defended); (3) the physical features of the nest and its immediate surroundings (e.g. its location within a hedge, park or continuous woodland); (4) the urban landscape structure around the nest (e.g. local road density or extent of impervious surfaces in the surroundings); and (5) direct and indirect estimates of human activities and practices (e.g. access to dense Muslim colonies for reasons stated above, efficiency of waste management, or human density). Further details of the recorded variables and their ecological rationale are given in Table 1 and in [20].

**Table 1:** Variables measured during nest defense trials conducted at Black kite nests within the city of Delhi (India).

Variable	Description, rationale for use and predicted effect
Julian date	Julian date of nest inspection. Earlier laying raptors are often older or higher quality individuals with higher parental investments and were thus expected to be more aggressive [52].
Breeding stage	The breeding cycle was divided into five main stages: (1) pre- incubation; (2) incubation (3) nestlings younger than 15 days; (4) nestlings of 15–30 days; (5) pre-fledging: 30–48 days old nestlings; (6) post-fledging. We expected defense to vary by stage because avian nest defense often varies through the breeding season in conjunction with the growing survival probabilities of the offspring e.g. [33–35].
Previous visits	Number of previous nest checks by the research team. This variable was fitted to control for potential habituation or reinforcement of aggressiveness by repeated sampling of the same pair [53].
Team size	Number of people in the research team (2 or 3). This was fitted to examine the impact of the number of intruders on defense, if any.
Number of onlookers	Number of people (not belonging to the field-team) within 20 m of the nest during the defense trial. This was fitted to examine the impact of the number of onlookers on defense, if any.
Number of offspring	Number of eggs or chicks in the nest at the time of the defense trial. We expected higher aggression by pairs with larger parental investments, as found in some previous studies e.g. [33–35].
NND5 (m)	Mean distance to the five closest kite neighbors. This variable focused on the impact of local, spatial arrangement on defense intensity. We expected higher defense under more crowded conditions (i.e. at higher quality, more attractive sites, which may entail higher parental investments).
Territories within 200 m	Number of territories occupied within 200 m of the target nest. This variable focused on the impact of local density on defense intensity. We expected higher defense levels at higher local densities (i.e. at higher quality, more attractive sites, which may entail higher parental investments).
Colony size	Number of nests within the kite colony. We expected larger colonies to be more attractive to individuals of a semi-social species, or to be associated with higher vigilance and larger food supplies, leading to a higher motivation for defense.
Tree arrangement	Categorical variable: 1 = isolated tree/pylon; 2 = line of trees (e.g. along an avenue); 3 = parkland (scattered trees with > 5–10 m of open ground between them, typically grassland in urban parks); 4 = woodlot. These habitat configurations are known to be differentially attractive to Delhi kites [20] and were fitted in order to investigate links between habitat quality, urban landscape configuration and defense intensity.
Balcony	Categorical variable: 0 = absence, 1 = presence of a balcony within 20 m of the nest. We predicted that pairs in such close and constant contact with humans could show higher aggressiveness through habituation and loss of fear.
Index of road density	Number of asphalted roads crossed by a 500 m north-south and a 500 m east-west transect crossing each other on the nest. Delhi kites over-select areas with more extensive road networks, which are one of their main foraging habitats [20]. Thus, we expected defense-levels to increase with road density.
Urban cover	Percentage area covered by built-up structures (buildings, roads, parking lots, or any other impervious surface) within 500 m of the nest. Urban and tree cover were fitted to investigate links between offspring defense and urban landscape configurations. Urban cover was also fitted as a quadratic effect to test the “intermediate disturbance hypothesis” commonly proposed in the urban ecology literature [54], by which the favourability of urban ecosystems to wildlife peaks at intermediate levels of the urbanization gradient.
Green cover	Percentage area covered by shrub/tree vegetation within 500 m of the nest. Urban and tree cover were fitted to investigate links between offspring defense and urban landscape configurations.
Hygiene score	Level of sanitation: 1 = clean areas; 2 = areas under poor waste management regimes ^a^. The level of street sanitation is an important component of habitat quality for this population [20]. We expected higher aggression at sites with lower sanitation because of frequent exposure to humans and because larger food supplies may imply larger broods and thus higher parental investments.
Human density	Average number of people walking within 2m of a stationary observer during 5 min at 10 locations randomly plotted within 200 m of the nest ^b^. Delhi kites over-select sites with intense human activity in the streets, leading to more food in the form of human refuse [20]. We expected defense-levels to increase with human density in the streets because of frequent exposure to humans and because larger food supplies may imply larger broods and thus higher parental investments.
Access to Muslim subsidies	First component (PC1) of a principal component analysis on Muslim density and on the proximity to the three closest Muslim colonies (see Methods). Muslim subsidies are one of the main food resources for Delhi kites [20, 21] and ready access to them was predicted to boost offspring-defense because of frequent exposure to humans and because larger food supplies may imply larger broods and thus higher parental investments.

^a^ Categorical variable with two levels: 1 = efficient waste disposal with very scarce or no organic refuse in the streets; 2 = abundant and widespread refuse in the streets throughout the area, either in small frequent piles, in illegal ephemeral dumps, or as individual items scattered a bit of everywhere through all streets [20].

^b^ Counts were only operated between 10:00–17:00 hrs and avoided during atypical, momentary peak periods of human traffic, such as exits from work or schools, in order to maintain consistency across sites [20].

In particular, a key variable in our previous analyses on the predictors of kite site selection, occupancy and breeding performance was the ease of access to dense Muslim colonies, which provide abundant food supplies in the form of ritual subsidies [20]. More specifically, we previously showed that Delhi kites over-selected for breeding sites closer than available to the 1st, 2nd and, possibly, 3rd nearest Muslim colony (see [20] for details). Thus, to provide a comprehensive measure that integrated the proximity to the three nearest Muslim colonies with their human population density (under the assumption that higher rates of refuse and ritualized-feeding should occur in more densely-populated Muslim colonies), we extracted the first component of a PCA [26] run on these four aforementioned variables. Its PC1 (hereafter “access to Muslim subsidies”) explained 65% of the variance and had a high positive loading on Muslim population density and high negative loadings on the distance to the 1st, 2nd and 3rd closest Muslim colonies. Thus, it provided an increasing index of access to abundant “Muslim subsidies”.

### Statistical analysis

We employed a linear mixed effect ordinal regression (cumulative-link mixed effect model), [27, 28] through package “ordinal” [29] to examine the effect of environmental, urban and human variables on the ordinal score of offspring defense. The ordinal regression was run on 657 defence trials conducted at 101 unique nesting territories. Because territories were sampled repeatedly, and because territories within the same plot were closer and thus potentially more similar than territories sampled in different plots, we fitted as a random effect territory identity nested within plot identity and year, so as to control for pseudoreplication and spatial autocorrelation [30]. To reduce collinearity and the number of variables presented to the logistic regression, we considered pairs of strongly inter-correlated variables (r > 0.60) as estimates of a single underlying factor, and only retained for analysis the one estimated to be more biologically important for the study organism.

To explore further the potential link between defense intensity and subsequent breeding benefits, we related the eventual number of fledged young to the intensity of defense during incubation (i.e. about two months before fledging) by means of a linear mixed model, again with territory identity nested within plot identity and year as a random term.

All multivariate models were built through a backward stepwise procedure following Zuur et al. [30]: all explanatory variables were fitted to a maximal model, extracted one at a time from the maximal model, and the associated change in model deviance was assessed by the significance of a likelihood-ratio test; the procedure was repeated until we obtained a final model which only included significant variables [30]. To avoid over-parameterization, we ensured never to fit more than N/3 variables to each maximal model, where N is the sample size of the analysed dataset [31]. Interactions were fitted only when we had a priori hypotheses about their potential effect, based on our field observations and knowledge of the population. To explore the possibility of curvilinear relationships, we fitted continuous variables as linear and also as quadratic terms. Explanatory variables were fitted as standardized Z-scores because of their different measurement units and in order to better evaluate their relative importance [32]. Model assumptions were checked by investigating QQ plots, histograms of residuals, and plots of standardized and normalized residuals against fitted values and against explanatory variables [30, 31]. All mixed models were implemented in R.3.4.3 [33], all tests are two-tailed, statistical significance was set at < 0.05, and all means are given ± 1 SE.

## Results

**Fig 1:**
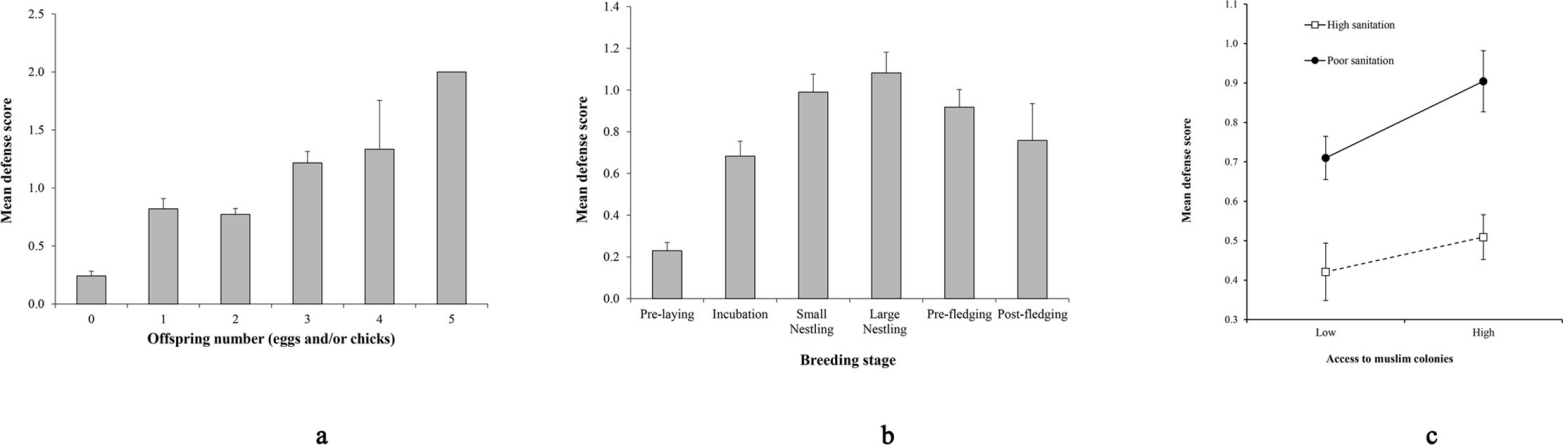
The intensity of offspring defense by kites in Delhi (India) varied with: panel (a) the stage of the breeding cycle; panel (b) the number of offspring (eggs and/or chicks) in the nest at the time of the defense trial; and panel (c) the interaction between access to Muslim subsidies and the amount of human waste in the streets (the black circles and the solid line indicate breeding sites with poor street sanitation, while the white quadrats and dotted line portray cleaner sites with less refuse in the streets). In panel c, Access to Muslim subsidies is shown above and below the median value (“high” and “low”, respectively) for clarity of presentation. Error bars represent ± 1 SE.

Several variables entered the mixed model ordinal regression (Table 2). First, defense intensity increased progressively along the breeding cycle and then declined in its final stages, especially after the young fledged from the nest (Fig 1A). Second, defense levels increased with the number of offspring in the nest (Fig 1B). Third, the interaction of Access to Muslim subsidies and Hygiene score was also significant (Table 2 and Fig 1C): under conditions of poor sanitation, defense levels increased more steeply with access to dense Muslim colonies than under cleaner conditions, suggesting that low sanitation and ready access to Muslim subsidies acted in concert, i.e. synergistically affecting aggressiveness. Fourth, defense intensity declined with the green cover and was minimum at intermediate levels of built-up cover (Table 2A).

**Table 2:** Cumulative-link mixed effect ordinal regression (a) testing the effect of environmental, urban and human variables on the ordinal intensity of offspring defense; and (b) linear mixed effect model testing the effect of intensity of offspring defense in incubation on eventual fledgling production.

Variable	ß ± SE	Z-test	*P-* value
**a. Dependent variable: Intensity of defense (N = 657)** ^a^^,^^b^			
Breeding Stage (incubation)	1.11 ± 0.8	1.39	0.164
Breeding Stage (small nestling)	3.11 ± 0.82	3.81	< 0.001
Breeding Stage (large nestling)	3.83 ± 0.8	4.78	< 0.0001
Breeding Stage (pre-fledging)	2.34 ± 0.76	3.07	0.002
Breeding Stage (post-fledging)	0.71 ± 0.97	0.73	0.463
Offspring number	1.61 ± 0.33	4.81	< 0.0001
Access to Muslim subsidies	-6.23 ± 2.01	-3.1	0.001
Hygiene score	3.25 ± 1.01	3.24	0.001
Access to Muslim subsidies * Hygiene score	4.5 ± 2.02	2.22	0.026
Green cover	-1.65 ± 0.68	-2.4	0.016
Urban cover	-3.69 **±** 1.62	-2.28	0.022
Urban cover ^2	3.20 ± 1.67	1.9	0.057
**b. Dependent variable: fledglings produced** ^c^ **(N = 103)**			
Intensity of defense (during incubation)	0.28 ± 0.12	2.27	0.023
Intercept	-0.36 ± 0.17	-	-

^a^ Cumulative link mixed model with a logit link function, based on N = 657 defense trials from 101 independent territories. The dependent variable is the ordinal score of offspring defense intensity. Territory-identity nested within plot-identity and year was fitted as a random factor.

^b^ Variables presented to the model: Julian date, Team size, Number of onlookers, Previous visits, Breeding Stage, Offspring number, NND5, Territories within 200 m, Tree arrangement, Balcony, Index of road density, Urban cover, Green cover, Hygiene score, Human density, Access to Muslim subsidies, Access to Muslim subsidies*Hygiene score, Access to Muslim subsidies*Urban cover, Access to Muslim subsidies*Green cover (the rationale for fitting interactions can be found in the Methods).

^c^ Generalised linear mixed model with Poisson errors and a logit link function, based on N = 103 defense trials from 60 independent territories sampled during incubation. The dependent variable is the number of young raised to fledging stage. Territory-identity nested within plot-identity and year was fitted as a random factor.

**Fig 2:**
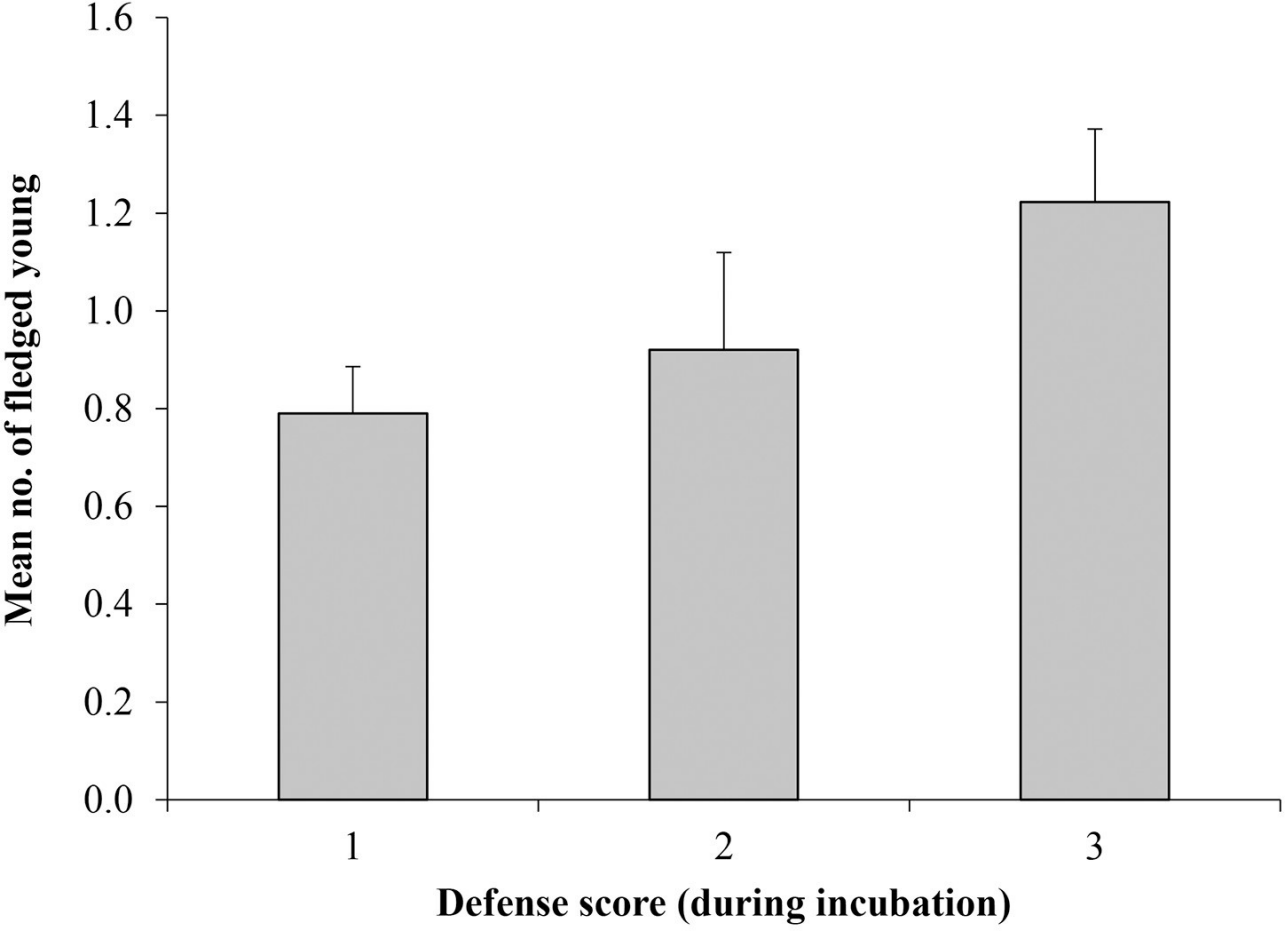
Number of fledglings produced by a pair in relation to the defense intensity exhibited by that pair two months earlier during incubation. Error bars represent ± 1 SE.

Finally, the number of fledglings produced by a pair was positively related to the defense intensity recorded for the same pair about two months earlier during incubation (Table 2B and Fig 2).

## Discussion

Kite defense levels progressively increased through the breeding cycle and reflected the number of offspring in the nest during each trial. These results confirmed those of several earlier studies e.g. [34–36] and suggested that parents tuned their defense response in relation to their parental investment, i.e. on the quantity and future survival prospects of their offspring, which increased through the breeding cycle. The fact that defense intensity early in the season predicted eventual young production months later, by the end of breeding, implied three non-exclusive possibilities: (1) parents could estimate the eventual likelihood of breeding success early in the season and set their defense accordingly; (2) aggressive nest defense lowered predation rates at the nest, with consequent benefits for young production; or (3) high quality individuals (e.g. healthier, or larger) were simultaneously more aggressive and better breeders, generating a positive association between two parameters separated by months in time. For example, kites that were more aggressive against humans could potentially be more aggressive against other more common nest predators such as crows or monkeys [21]. In support of this idea, in another study, nest defense by a falcon was experimentally shown to lower nest predation rates by corvids [35]. The above mix of associative and causative mechanisms produced results that are typical of avian nest defense studies e.g. [37, 38], suggesting that life in an urban setting did not disrupt the typical links between behavioural traits and vital rates found in avian populations.

More notably, despite constant close exposure to people, kite defense suggested that humans were not perceived as a neutral component of the urban landscape, but rather as a potential danger when they approached a nest. This implied a capability by kites to discriminate human attitudes and adjust their behaviour in a context-dependent manner, approaching people to very close quarters for feeding but fleeing and sometimes even attacking them when defending their offspring. Furthermore, defense levels varied through the city in relation to cultural ritual-feeding practices, refuse management and landscape composition. In particular, defense intensity was higher at sites that combined ready access to dense Muslim colonies (where kites are fed by humans by tossing meat-scraps at very close quarters) with poor sanitation (which promotes frequent feeding on anthropogenic waste close to people, e.g. at ephemeral garbage dumps also used by poor rag pickers digging for useful materials). This spatial association could be the result of three non-exclusive mechanisms: (a) frequent and reiterated, close contact with humans may have lowered fear, thus enhancing boldness and aggression; (b) sites close to ritual-feeding areas or with poor sanitation are over-selected by kites and thus likely occupied by higher quality individuals with higher parental investments [20], leading to higher defense intensity; and (c) bolder individuals may be more likely to withstand constant close proximity to humans and a bolder temperament is associated with greater aggression in some species e.g. [39]. Thus, individual quality, personality, habituation and motivation may have generated a spatial association between a behavioural strategy and a human cultural landscape, thus contributing to the growing appreciation of the importance of human cultural geographies for urban ecology e.g. [40–43].

While the exact mechanism remains uncertain, the behavioural response of kites to a perceived threat was finely tuned on the spatial arrangement of human activities and ritual practices, their consequent attitudes towards the birds, and their waste management organization. In turn, this would create a dynamic behavioural landscape, reflecting the underlying urban mosaic of resources, structures and human attitudes, to which kites will necessarily have to adapt and respond, as shown for species that colonized urban environments more recently [5].

The fact that aggression peaked at close human proximity suggested that close coexistence and habituation to people led to a loss of fear and heightened boldness towards humans, rather than an enhanced capability to avoid them by keeping a “low profile” or learning to ignore them. Such dynamics may have been further favoured by the generally positive, religiously-based attitudes of Indian people towards wildlife, as reported by several studies e.g. [44–46].

Overall, these results confirm and extend earlier findings of more aggressive offspring defense by urban than rural individuals of a given species e.g. [47–49], suggesting that the route to close coexistence with humans is often accompanied by fine-grained, context-dependent strategies and trade-offs, rather than evolution of “blind tolerance” and indifference towards human activities [50]. In this sense, most animals making frequent contact with humans (through colonization of urban habitats, or through peri-urban encroachment) will likely need to develop cognitive capabilities and behavioural tactics that will enable them to exploit humans and cope with their omnipresent disturbance rather than learning to ignore them, in order to attain long-term coexistence e.g. [10, 51]. In turn, acquisition of such traits will likely be shaped by a two-way interaction between human perceptions, attitudes and practices on one part and daily experience and habituation to humans on the part of the animal. For synurbic species, like kites, at the high end of the spectrum of adaptation to an urban life, the above cited interaction may lead to behavioural and demographic traits fine-tuned not only on urban physical structures, but also on human cultural practices and attitudes, which for many species may become the most important, defining dimensions of their urban niche.

To date, most studies of animal behavioural responses to urbanization have focused on the comparison between urban and rural individuals, in order to draw inferences on the characteristics that enable or mediate the colonization of highly anthropogenic urban environments e.g. [7, 9, 10]. Here, we show that marked heterogeneity in behavioural responses to humans also continue to exist within cities and after centuries of initial urban colonization, suggesting further fine-tuning of behavioural traits on specific dimensions of the urban environment. In this sense, the urban-rural comparison does not target the end-result of colonization, but rather defines only the beginning of a hierarchical process of adaptation to humans, who are increasingly concentrated in cities. Thus, more research is needed on the fine-grained adjustments to urban structure and human culture by animals that are already in their mature stage of adaptation to an urban life.
